# Effect of Salt Solution Erosion on Mechanical Properties and Micropore Structure of Recycled Fine Aggregate ECC

**DOI:** 10.3390/ma17112498

**Published:** 2024-05-22

**Authors:** Yuanhang Xiang, Fengxia Han, Qing Liu

**Affiliations:** 1 School of Architectural Engineering, Xinjiang University, Urumqi 830046, China; 107552104184@stu.xju.edu.cn (Y.X.); liuqing2666@xju.edu.cn (Q.L.); 2 Key Laboratory of Building Structure and Seismic Resistance of Xinjiang, Urumqi 830017, China

**Keywords:** engineered cementitious composite, recycled fine aggregate, salt solution erosion, mechanical properties, pore microstructure

## Abstract

This study examined the impact of sulfate and sulfate–chloride dry–wet cyclic erosion on the mechanical properties and microscopic pore structure of engineered cementitious composite (ECC) with recycled fine aggregate (RA). Uniaxial tensile tests and four-point bending tests were conducted to evaluate the mechanical properties of RAECC, while the resonance frequency ratio was used to assess the integrity of the specimens. Finally, X-ray computed tomography (X-CT) reconstruction was employed to analyze the erosion effects on the microscopic pore structure. The results showed that the uniaxial tensile strength and flexural strength of the RAECC specimens in corrosive solution first increased and then decreased, and the 5% Na_2_SO_4_ solution caused the most serious erosion of the specimens. The resonance frequency ratio of the specimens reached the peak value when they were subjected to dry–wet cycles 15 times in the 5% Na_2_SO_4_ solution. During the erosion process, the pore space of the specimen first decreased and then increased, and the number of pores increased. The erosion process is the result of the erosion products continuously filling and eventually destroying the pores, and the erosion damage produces a large number of new pores and poor sphericity, leading to a decline in mechanical properties.

## 1. Introduction

Concrete is widely used in construction, but concrete is a brittle material, prone to produce cracks via tensile cracking, which provide a pathway for water and salt ions to erode the infrastructure, leading to the permanent deterioration of structural elements and affecting their service life [1,2,3]. Since the ability to control the crack width of concrete is an important indicator of the durability of a structure, enhancing the crack control ability of concrete is essential to improving the durability of the structure. Engineered cementitious composite (ECC), which is made by incorporating fiber into the cement matrix, exhibits high strain-hardening properties and good ductility under load, with restricted crack widths during damage [4,5,6,7]. It can limit the penetration of aggressive ions, thus significantly improving the corrosion resistance of concrete.

In recent years, the overconsumption of natural sand resources and the disposal of large quantities of construction and demolition (C&D) waste have become a major concern. To address this issue, recycling C&D waste and converting it into recycled fine aggregate has become an important method to reduce concrete costs, alleviate the scarcity of natural resources, and improve environmental sustainability [8,9,10,11]. Previous research has shown that incorporating up to 40% recycled fine aggregate into concrete can yield good mechanical and durability properties [12,13,14]. This suggests the potential to create recycled fine aggregate (RA) from recycled construction and demolition materials and use it in ECC to produce RAECC. However, recycled aggregates usually have high porosity and water absorption [15,16], which leads to an increase in the permeable porosity and water absorption of RA-containing ECCs [17,18], properties that can seriously affect the mechanical and durability properties of concrete [19,20,21]. Therefore, it is crucial to investigate the impact of erosion on the microscopic pore structure changes of RAECC and its effect on mechanical properties.

The existing methods for the study of ECC pore structure mainly rely on the mercury intrusion porosimetry (MIP) technique to detect the distribution of the number of ECC pore sizes and volumes [22,23]. However, there are defects in the MIP detection technique, which cannot analyze the distribution of pores in three-dimensional space, and when mercury is pressed into it, it can damage the pore structure and cause high porosity, which leads to inaccurate data results [24]. X-ray computed tomography (X-CT) as an advanced non-destructive testing method solves this problem well. Soliman et al. utilized X-CT technology to investigate the impact of freeze–thaw cycles on the pore structure of asphalt concrete mixtures. They observed that with an increase in the number of freeze–thaw cycles, there was a corresponding increase in the total area of pores [25]. Liu et al. noted that erosion products initially filled the internal pores of concrete, leading to the formation of new cracks in later stages. This accelerated sulfate erosion, increased porosity, and introduced a method for the local deterioration assessment of concrete structures using X-CT and ultrasonic velocities [26]. Wang et al. employed X-CT technology to discover that during ECC sulfate dry–wet cycle erosion, small pores transitioned into larger pores with the accumulation of corrosion products, which was identified as the primary cause of concrete deterioration [27]. X-CT technology is capable of reconstructing intricate information such as the shape and distribution of pores in three-dimensional space, aiding in the examination of internal structural changes in RAECC following erosion [28,29,30,31,32,33]. By integrating these findings with the alterations in mechanical properties, a comprehensive understanding of the degradation of RAECC during salt solution erosion mechanisms can be achieved.

This study focuses on utilizing construction solid waste to create recycled fine aggregate replacing a portion of natural sand, for the production of RAECC. The research investigates the effects of salt solution erosion (sulfate and sulfate–chloride) on the mechanical properties and micropore structure of RAECC under dry–wet cycle conditions. The methodology includes testing the mechanical properties of eroded RAECC, utilizing resonance frequency tests to assess specimen integrity, and employing X-CT microscopic inspection (voxel resolution of 4.9μm) to analyze pore structure evolution post-erosion. The integration of microscopic and macroscopic analyses elucidates the degradation mechanism of RAECC when exposed to dry–wet cycle erosion by salt solutions.

## 2. Materials and Methods

### 2.1. Material Properties and Mix Proportion Design

**Figure 1:**
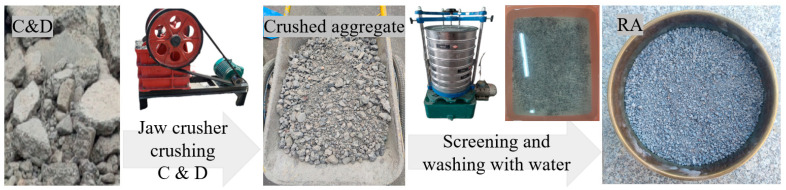
Recycled Fine Aggregate Preparation.

**Figure 2:**
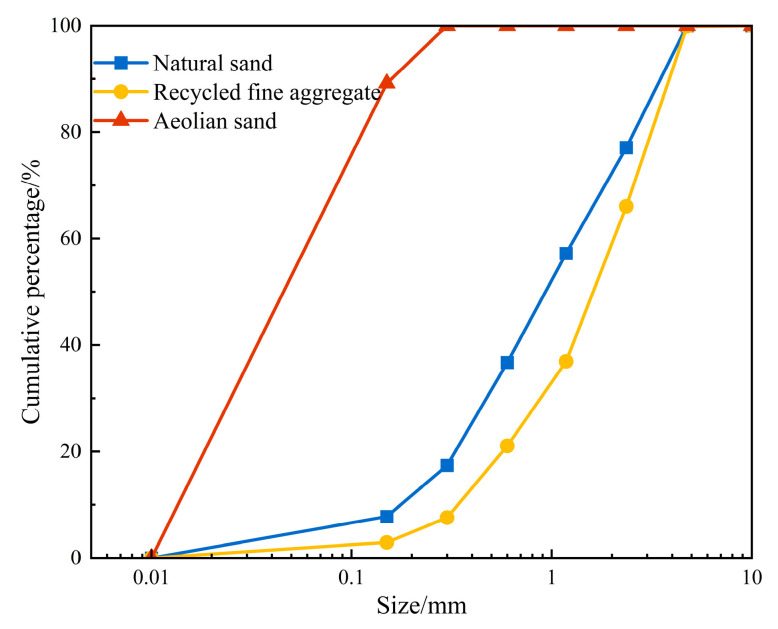
Cumulative curve of grain size grading.

The experiment used P•O 42.5R ordinary Portland cement from Xinjiang Tianshan Cement Company (Urumqi, China). Class F fly ash (grade II) from China Construction Western Construction Xinjiang Co. (Urumqi, China)was employed. Natural sand, aeolian sand sourced from Kumutag in Xinjiang, and laboratory-created recycled fine aggregate (RA) were chosen as fine aggregates. The production process of RA is illustrated in Figure 1, with a water absorption rate of 9.4%. Additionally, the particle size gradation of the three fine aggregates can be seen in Figure 2. A polycarboxylic-acid-based high-performance water-reducing agent demonstrating a 25% water reduction rate and 36% solid content was used. A thickener and defoamer supplied by Qingjun Chemical (Jinzhou, China) were incorporated. For the production of ECC, polyethylene (PE) fiber of model 1600D developed by Telif Fiber Manufacturing Factory (Laiwu, China) was chosen, with specific performance indexes detailed in Table 1. Analytically pure anhydrous sodium sulfate from Zhiyuan Chemical Factory (Tianjin, China) and analytically pure sodium chloride from Sinopharm Chemical Reagent Co., Ltd. (Shanghai, China) were used. A modified mix proportion, determined through orthogonal testing on the original mixing ratio [34], indicated that 40% and 20% of recycled fine aggregate and wind-logged sand were replacements for natural sand. The volume mixing amount of PE fiber was set at 1.5% vol. Specific mix proportions can be seen in Table 2.

### 2.2. Specimen Design

**Figure 3:**
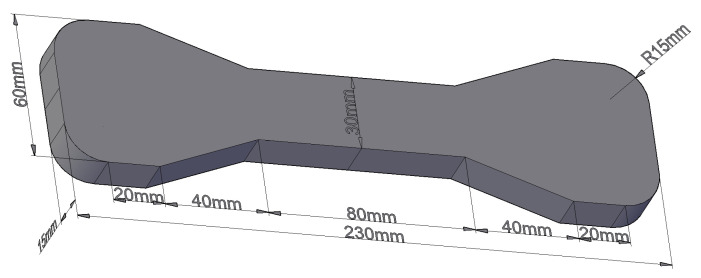
Bone-type specimen schematic diagram.

RAECC bone-type specimens (Figure 3) with dimensions of 230 mm × 60 mm × 15 mm were fabricated for uniaxial tensile testing, and RAECC prismatic specimens with dimensions of 400 mm × 100 mm × 100 mm were fabricated for resonance frequency determination.

### 2.3. Experimental Procedures

#### 2.3.1. Dry–Wet Cycle Test Plan

**Figure 4:**
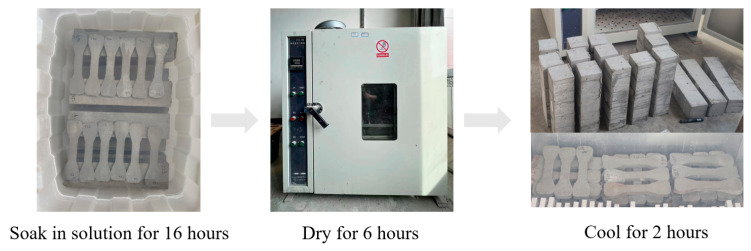
Schematic diagram of dry–wet cycle.

Referring to the specification GB/T 50082-2009 [35], the chemical erosion of specimens under dry–wet cycles was conducted on RAECC. A total of 40 dry–wet cycles were completed, with mechanical properties being tested once every 10 cycles. The dry–wet cycle erosion test procedure is illustrated in Figure 4. Specimens that had been through 28 days of regular upkeep were soaked in a solution for 16 h, then taken out and put in a lab oven adjusted to (80 ± 5 °C) for dehydration for 6 h, succeeded by 2 h of cooling, resulting in a dry–wet cycle every 24 h. The dry–wet cycle solutions included aqueous solution (W), 5% Na_2_SO_4_ solution (S), and 5% Na_2_SO_4_—3% NaCl solution (F).

#### 2.3.2. Loading Scheme for Uniaxial Tensile Test Plan

**Figure 5:**
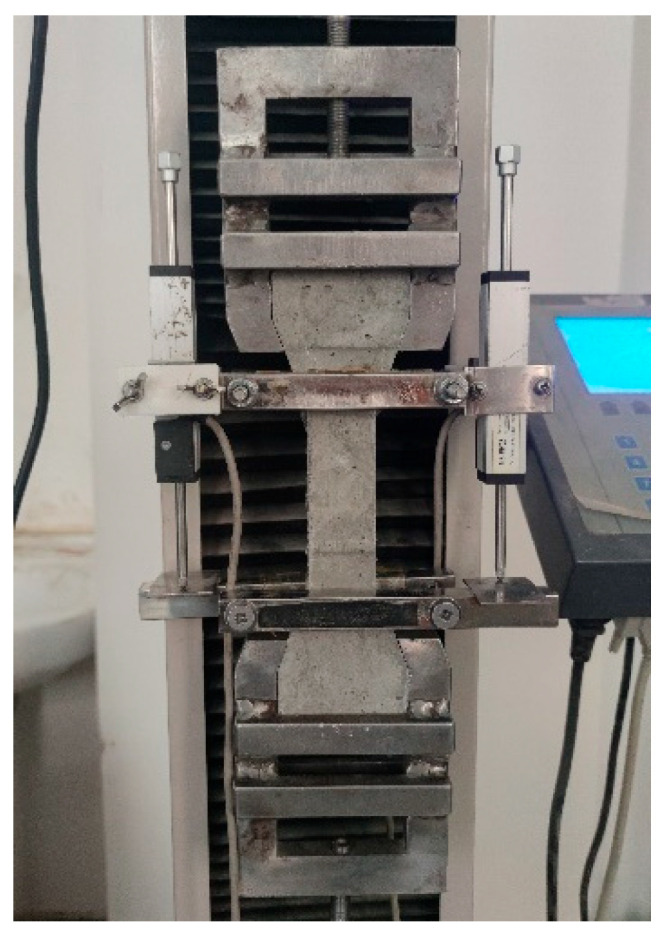
Tensile testing equipment.

After the bone-type specimens were cured to the set age, the uniaxial tensile test of RAECC was carried out by a 5 kN LCD electronic tensile machine of model LDS-5 (Figure 5). The displacement control was adopted, and the loading speed was 0.5 mm/min. The changes in the length of the monitoring zone were recorded by installing two sets of Linear variable displacement transducers (LVDT) on both sides of the specimen, and the final value of the length change was taken as the average value of the two test results, with a measuring scale distance of 80 mm. Before the tensile test, pre-stretching was carried out, the tensile force data on the instrument of the tensile testing machine were observed, and when the data were displayed as 100 N, the recording of the test data was started to ensure that the specimen did not slide in the tensile process.

#### 2.3.3. Four-Point Bending Test Plan

**Figure 6:**
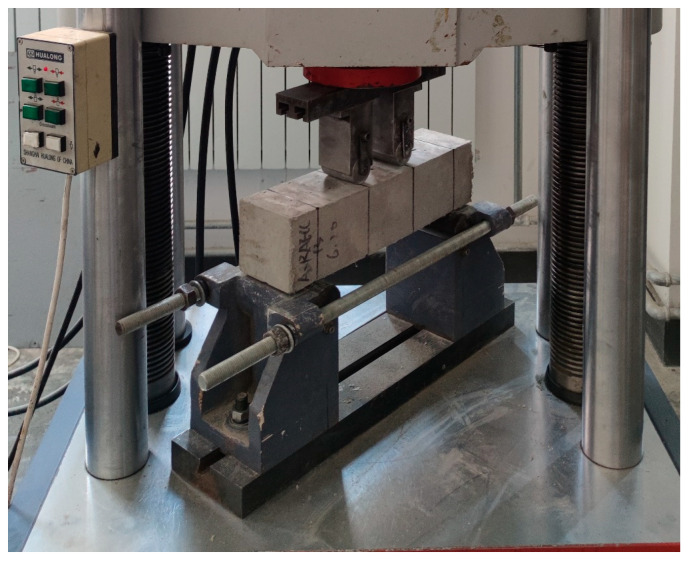
Four-point bending test device.

After the prismatic specimens were cured to the set age, the four-point bending test of RAECC (Figure 6) was carried out by the microcomputer-controlled electro-hydraulic servo universal testing machine (WAW-600, range 600 kN) produced by Shanghai Hualong Testing Instrument Co. The prismatic specimens were placed on the lower two rollers at 50 mm from each edge of the specimen on both sides, and the loading method was stress-controlled with a loading speed of 0.06 MPa/s until the specimen was damaged by bending.

#### 2.3.4. Resonance Frequency Detection Test Plan

**Figure 7:**
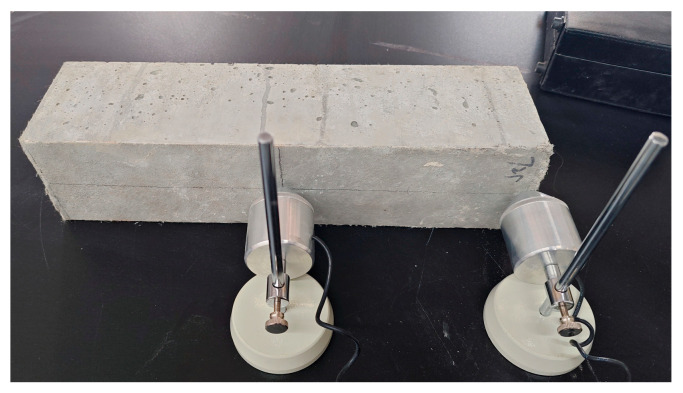
Resonance frequency detection.

Referring to the specification GB/T 50082-2009 [35], as shown in Figure 7, the fixed side of the RAECC prismatic specimens was selected for the resonance frequency test, three specimens were tested in each group, and the test was carried out once every 5 dry–wet cycles. Since the initial resonance frequency of each group of specimens was not the same, to facilitate the comparison of the resonance frequency change between different groups of specimens, the change in resonance frequency ratio was used for the comparison, which is calculated as shown in Equation (1):

(1) $$R_{f}=F/F_{v}$$

where $R_{f}$ represents the resonance frequency ratio, *F* represents the average value of resonance frequency of specimens after erosion, and $F_{v}$ is the average value of resonance frequency of specimens of the same age without erosion.

#### 2.3.5. X-CT Detection Scheme

The X-CT scan was conducted at room temperature using a DS600/225F100 imaging system. The scanning voltage was set to 130 kV, with a current of 160 μa, an exposure time of 500 ms, and a voxel resolution of 4.9 μm. A total of 940 projections were captured in the CT scans, with a spacing of 4.9 μm between each projection. Sampling and scanning of a bone-type specimen in 5% Na_2_SO_4_ solution, which was subjected to severe erosion by the salt solution, was carried out by removing a cube of approximately 4.6 mm × 4.6 mm × 4.6 mm from the middle of the bone-type specimen, and due to the small size of the specimen sampled for examination, grinding was used to achieve a fixed size.

## 3. Results and Discussion

### 3.1. Effect of Erosion on the Mechanical Properties of RAECC

#### 3.1.1. Tensile Stress–Strain Curve

**Figure 8:**
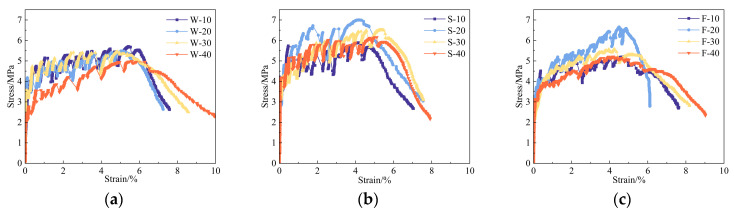
RAECC tensile stress–strain curve: (**a**) aqueous solution (represented by W), (**b**) 5% Na_2_SO_4_ solution (represented by S), and (**c**) 5% Na_2_SO_4_—3% NaCl solution (represented by F).

Figure 8 shows the tensile stress–strain curves of the RAECC bone-type specimens in different salt solutions after 10, 20, 30, and 40 cycles of dry–wet erosion (numbering is denoted by x-y, with x being the solution and y being the number of dry–wet cycles). It can be seen that the tensile curves of the RAECC specimens in different solutions show obvious hardening characteristics with the increase in erosion cycles. However, the specimens in 5% Na_2_SO_4_ solution and 5% Na_2_SO_4_—3% NaCl solution were brittle due to the erosion-induced matrix, and the erosion made the ductility lower than that of the specimens in aqueous solution.

#### 3.1.2. RAECC Tensile Performance

**Figure 9:**
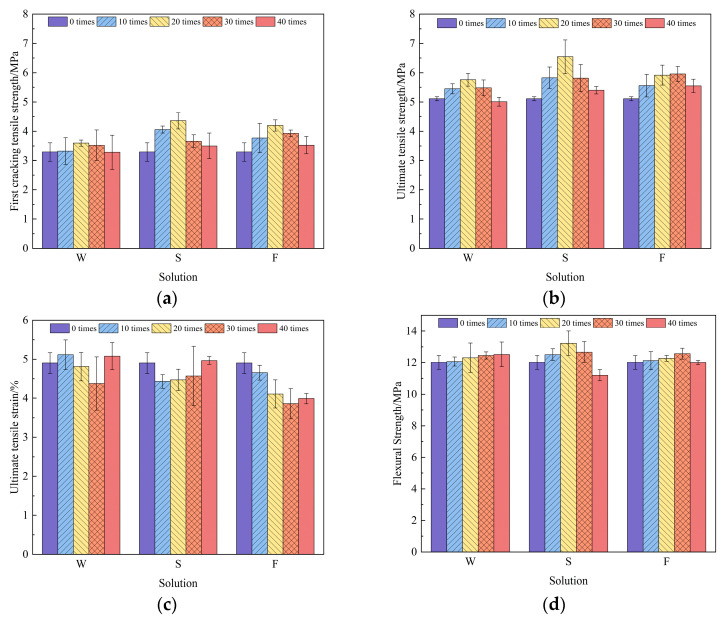
Mechanical properties of RAECC: (**a**) first cracking tensile strength, (**b**) ultimate tensile strength, (**c**) ultimate tensile strain, and (**d**) flexural strength. The codes W, S, and F in the figure denote an aqueous solution, a 5% Na_2_SO_4_ solution, and a 5% Na_2_SO_4_—3% NaCl solution, respectively.

The uniaxial tensile test results of the RAECC bone-type specimens after dry–wet cyclic erosion in different solutions are shown in Figure 9a–c. It can be seen that the ultimate tensile strength of the specimens eroded in the three solutions showed an increasing and then decreasing trend with the increase in cycles, generally higher than before erosion. The peak strength of the specimens in aqueous solution and 5% Na_2_SO_4_ solution appeared after 20 dry–wet cycles, and the peak strength of the 5% Na_2_SO_4_—3% NaCl solution appeared after 30 dry–wet cycles. After 40 dry–wet cycles, the ultimate tensile strength of the specimens in aqueous solution, 5% Na_2_SO_4_ solution, and 5% Na_2_SO_4_—3% NaCl solution decreased by 2.0%, increased by 5.7%, and increased by 8.6%, respectively, compared with the non-eroded specimens.

The order of erosion severity of the RAECC specimens by the different salt solutions was 5% Na_2_SO_4_ solution > 5% Na_2_SO_4_—3% NaCl solution > aqueous solution, of which the 5% Na_2_SO_4_ solution specimens exhibited the most obvious trend of decline during the erosion cycle in terms of ultimate tensile strength, with a difference of 1.15 MPa between the maximum value and the minimum value. This is due to the existence of SO_4_^2−^ in the 5%Na_2_SO_4_ solution, resulting in a large number of erosion products (such as AFt) being generated. Erosion products can fill the matrix at the beginning of the erosion, but as the erosion continues, excessive erosion products will destroy the original pore structure, resulting in the formation of new matrix defects, causing a reduction in strength. In the 5%Na_2_SO_4_—3%NaCl solution specimens, due to the reaction of Cl^−^ and SO_4_^2−^, which robs the solution of SO_4_^2−^ via the generation of Friedel’s salt [36], reduces the amount of AFt generated, and retards the erosion of the RAECC matrix by SO_4_^2−^, the effect of 5% Na_2_SO_4_—3% NaCl solution erosion on strength was stronger than that of the aqueous solution but weaker than that of the 5% Na_2_SO_4_ solution [37].

The first cracking tensile strength and ultimate tensile strength had the same trend, increasing with the increase in ultimate tensile strength. Contrary to the trend of ultimate tensile strength, the ultimate tensile strain showed a trend of decreasing and then increasing; however, the strain of the specimens in the 5% Na_2_SO_4_ and 5% Na_2_SO_4_—3% NaCl solutions under the same tensile strength was lower than that of the aqueous solution specimens, which indicates that the erosion of salt particles caused the matrix to become brittle, thus causing the strain to decrease.

#### 3.1.3. RAECC Flexural Strength

The four-point bending test results of the RAECC prismatic specimens after dry–wet cycle erosion in different solutions are shown in Figure 9d. It can be seen that the flexural strength of the specimens in the aqueous solution increased with the increase in cycles, and the flexural strength of the specimens in the 5% Na_2_SO_4_ solution and 5% Na_2_SO_4_—3% NaCl solution increased and then decreased. The erosion of the specimens in the 5% Na_2_SO_4_ solution was severe, and the strength decreased by 4.6% compared with that of the uneroded specimen after 40 dry–wet cycles. The order of erosion severity of the RAECC specimens by the different salt solutions was 5% Na_2_SO_4_ solution > 5% Na_2_SO_4_—3% NaCl solution > aqueous solution, and the results of the flexural strength of specimens in the erosive solutions were consistent with the ultimate tensile strength.

### 3.2. Effect of Salt Solution Erosion on the Microstructure of RAECC

#### 3.2.1. Effect of Erosion on Resonant Frequency of Specimens

**Figure 10:**
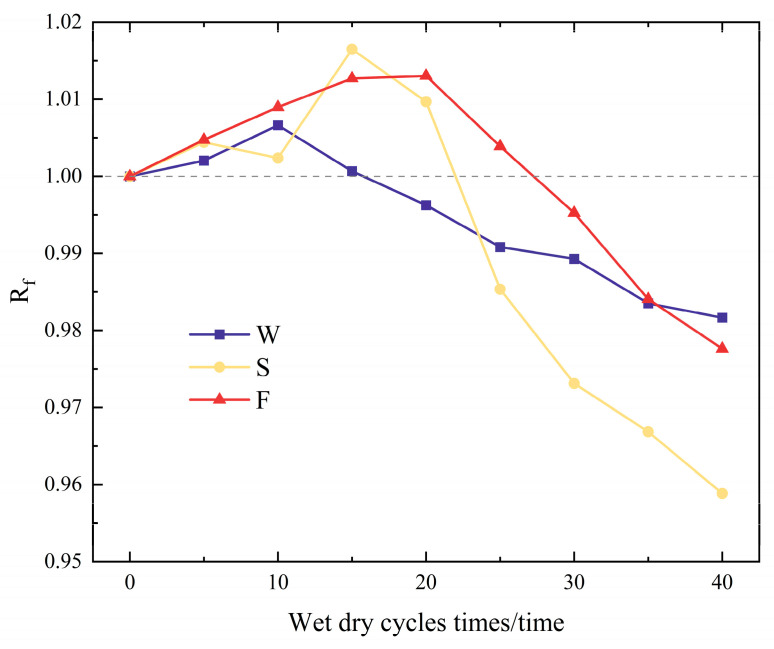
Resonance frequency ratio.

The resonance frequency of the RAECC prismatic specimens can indicate the level of internal structure integrity—the higher the integrity, the greater the resonance frequency [38,39]. After every five dry–wet cycles, three sets of resonance frequency data were gathered for the prismatic specimens. These values were then averaged and substituted into Equation (1). The results are shown in Figure 10. It was observed that the $R_{f}$ values of the RAECC prismatic specimens in all three solutions increased and then decreased. In the aqueous solution, the $R_{f}$ values of the specimens reached a peak after 10 cycles, while those of the specimens in the 5% Na_2_SO_4_ solution and the 5% Na_2_SO_4_—3% NaCl solution reached peaks after 15 and 20 cycles, respectively.

The filling effect of the erosion products inside the prismatic specimen caused the interior to become complete, increasing the $R_{f}$ value. However, with an increase in the number of cycles, the excessive erosion products started to damage the original internal pore structure, creating new matrix defects, which led to a decrease in completeness and, in turn, a decrease in the $R_{f}$ value. Based on the trend of the $R_{f}$ value, the order of erosion severity of the RAECC prismatic specimens by dry–wet cycling in different salt solutions was as follows: 5% Na_2_SO_4_ solution > 5% Na_2_SO_4_—3% NaCl solution > aqueous solution.

#### 3.2.2. Impact of Erosion on Matrix Pores

**Figure 11:**
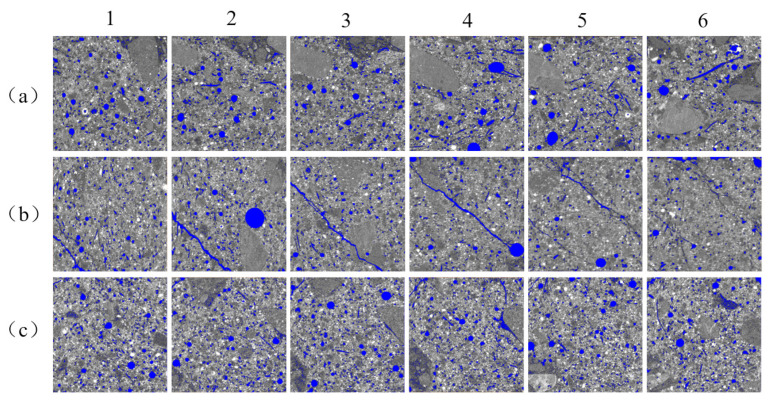
X-CT scanning 2D feature slices: (**a**) C, (**b**) S-20, and (**c**) S-40.

From the previous section, it is known that the 5% Na_2_SO_4_ salt solution eroded the specimens most severely; therefore, X-CT scans of the RAECC specimens after undergoing 0, 20, and 40 dry–wet cycles of erosion by 5% Na_2_SO_4_ salt solution were performed and analyzed (specimen numbers are denoted by C, S-20, and S-40, respectively.). The pore distribution of 2D slices of the X-CT scans of the specimens is shown in Figure 11, where blue color was chosen for rendering the pores, the characteristic slices in terms of increasing height are shown from left to right, and six images were selected from each group of specimens to be analyzed.

From Figure 11, it can be seen that the pore distribution in the 2D slices of the specimens is relatively uniform, and the number of pores and pore diameter of specimen S-20 is smaller than that of specimens in groups C and S-40, which is because after 20 dry–wet cycles, the erosion products filled the pores of the matrix, which reduced the pore diameter and the number of pores. After 40 dry–wet cycles, the overfilling of the erosion products damaged the matrix, which made the number of pores increase.

The light grey area of the cementitious material in Figure 11 is the freshly mixed cement paste containing fly ash, and the dark grey area of the cementitious material is the cement paste attached to the surface of the recycled fine aggregate. It can be seen that the number of cracks at the bond between the recycled fine aggregate and other materials in the specimen is higher than that of other fine aggregates, and the number of pores in and around the dark grey area is high. The high water absorption of the recycled fine aggregate and the internal pores and cracks generated during crushing led to an increase in the porosity of the specimen, and these cracks and pores provided transport channels for aggressive ions, thus affecting the durability of the specimen.

**Figure 12:**
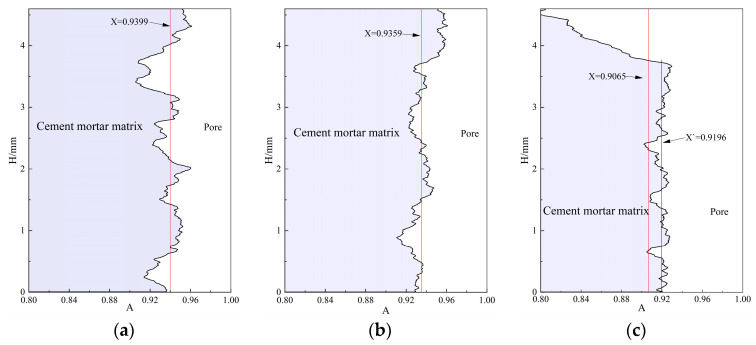
Change in cross-sectional area ratio along erosion depth: (**a**) C, (**b**) S-20, and (**c**) S-40.

Since different depths under the surface of the specimen are subjected to different degrees of erosion during sulfate erosion, and the closer the matrix is to the surface, the more serious the degree of sulfate erosion, the ratio of the area of the cement mortar matrix to the area of the overall cross-section of the CT-scanned 2D section (denoted as A) was calculated to analyze the effect of erosion on the internal porosity, and the specific results are shown in Figure 12. The straight line perpendicular to the x-axis in the figure is the mean value of A on different slices (X), and the 0~4.6 mm of the vertical axis scale represents the distance of the CT-scanned 2D slices from the surface of the specimen (H). Due to the small size of the CT specimen, the S-40 specimen caused the specimen to have missing corners when grinding, so the A-value curve decreased sharply at the position farther away from the surface of the specimen, which was ignored in the later analysis. From the two-dimensional section in Figure 11, the specimen of group S-20 was sampled in a poor location, and there was a diagonally cut crack on the inside, which had some influence on the analysis.

The A-value distribution curves of the RAECC specimens under different erosion cycles had different trends, and the downward depression on the curves is the location of large holes. The A-value curve of the uneroded Group C specimens has a larger magnitude of change along the longitudinal axis, while the A-values of the other two groups of specimens have a smaller magnitude of change, which is attributed to the fact that the erosion products filled up some of the pores, making the pore distribution uniform in the 2D slices. From the size of the x-value, with the increase in dry–wet cycle erosion, the x-value decreased by 0.43% and 1.74% in turn; the change is small, but the existence of internal penetrating cracks in the S-20 specimen led to the x-value of the S-20 group being larger than the actual value, so the actual change trend of the x-value with the cycle firstly decreased and then increased, which is in line with the change rule of the resonance frequency.

**Figure 13:**
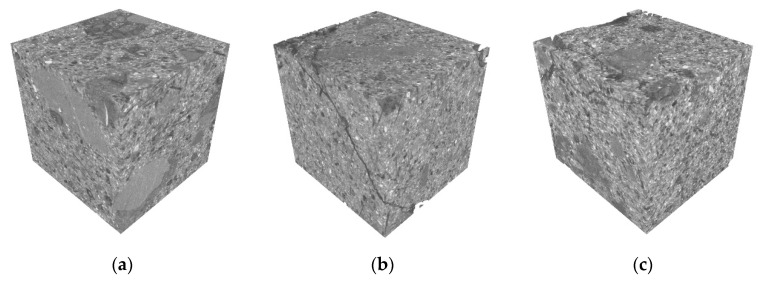
CT 3D reconstruction: (**a**) C, (**b**) S-20, and (**c**) S-40.

The X-CT scan 2D slice images were reconstructed in 3D as shown in Figure 13. It can be seen that after 40 sulfate dry–wet cycles, the integrity of the specimen was good and there was no obvious damage. The crack in Figure 13b was caused by sampling and was not an erosion-generated crack, and due to the presence of fibers, the crack occurred throughout the whole specimen, which was broken but did not shatter.

**Figure 14:**
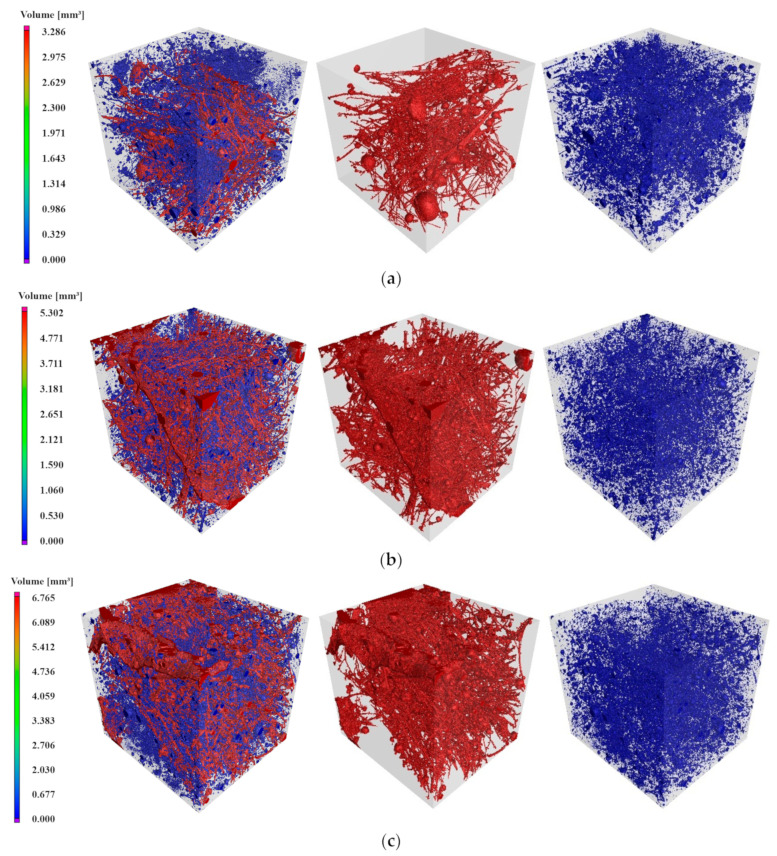
3D reconstruction of pore structure: (**a**) C, (**b**) S-20, and (**c**) S-40.

The pore structure of the 2D slices of the CT scan was analyzed after 3D reconstruction. The results, depicted in Figure 14, show red areas representing large, connected pores and blue areas indicating small pores. The analysis revealed that a significant portion of the total pores in the specimen were red connecting pores. This is attributed to the similarity in the grey scale between fibers and pores in the 2D slice images post-CT scanning, leading to the misidentification of PE fibers as pores during the 3D reconstruction. The PE fibers were well distributed in the specimen, often penetrating through most of the pores, resulting in a larger volume of maximum pores.

Smaller pores, on the other hand, were more uniformly distributed within the specimen, typically existing between aggregates and the grid formed by the largest connected pores. As the salt solution erosion progressed, the number of pores increased gradually. Larger gaps were filled with erosion products, transforming them into smaller pores, ultimately reducing the pore volume. The blue range in the 3D reconstruction map decreased after 20 dry–wet cycles. After 40 cycles, the original pore structure was disrupted by the transitional filling of erosion products, creating new pores and expanding the range of both red connecting pores and blue independent pores.

The specific pore space data of Figure 14 are presented in Table 3, indicating a continuous increase in the number of pores with erosion time. The increase was measured at 19.03% and 50.79%, respectively. This phenomenon was attributed to erosion products forming within the pores, causing larger pores to split into smaller ones, thereby increasing the overall pore count. Additionally, erosion led to substrate damage, creating new pores. The defect volume ratio in Table 3 characterizes the porosity inside the material, which can reflect the strength of the specimen to a certain extent. The defect volume ratio increased by 6.64% and 45.9%, respectively, with the increase in the number of wet and dry cycles, but due to the existence of internal penetrating cracks in the S-20 specimen and the small growth of the defect volume ratio from C to S-20, the actual defect volume ratio decreased with the increase in wet and dry cycle, followed by an increase, and the trend of the change was opposite to that of the mechanical properties. With the continuation of erosion, the defect volume ratio of the S-40 group reached 9.35%, causing a decrease in the mechanical properties.

The percentages of maximum porosity with respect to the total defects for the C, S-20, and S-40 specimens were 55.60%, 85.09%, and 74.40%, respectively, which accounted for a larger portion of the total defects in the specimens. To eliminate the influence of the largest defect on the pore data, the total volume of pores other than the largest defect volume was calculated as 2.624 mm^3^, 0.929 mm^3^, and 2.328 mm^3^, respectively. This volume decreased and then increased with the number of dry–wet cycles, decreasing by 64.6% and then increasing by 250.6%.

The refinement of the pore structure had a positive effect on the mechanical and durability properties of the specimens [40,41]. When the specimen underwent erosion and erosion products were generated, there was enough space inside the specimen to store these erosion products after 20 cycles of dry–wet cycling. The filling of the erosion products divided the pores into small cavities, leading to an increase in the number of pores but a decrease in the pore volume, resulting in specimen strength enhancement. After 40 dry–wet cycles, the erosion products continued to be produced, there was insufficient space inside the specimen to store the erosion products, and the salt-induced expansion stresses exceeded the tensile stress limit of the matrix. This resulted in the rupture of the original pore structure, leading to the generation of new pores, and consequently, both the number of pores and pore volume increased, resulting in the specimen strength decreasing.

**Figure 15:**
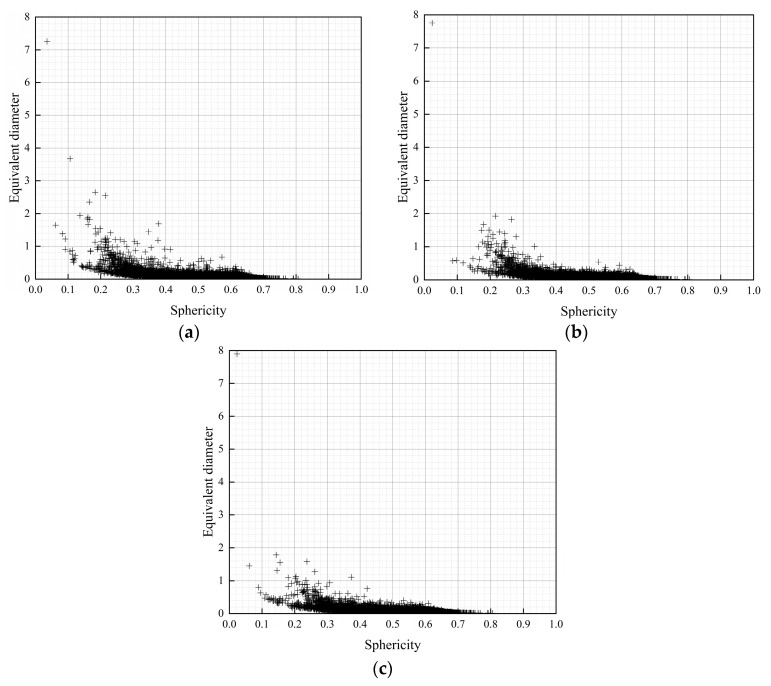
Pore sphericity: (**a**) C, (**b**) S-20, and (**c**) S-40.

The sphericity can reflect the geometry of the pores: with a sphericity close to 1, the shape of the pores approximates to a sphere; with a sphericity close to 0, the shape of the pores approximates to a crack. The distribution of the pore sphericity and the equivalent diameter of the pores obtained in the X-CT reconstruction are shown in Figure 15. It can be seen that the diameter of the pores in the uneroded control specimen was larger, the sphericity was smaller, and the shape of the pores in the original state was more similar to a crack. After 20 dry–wet cycles with erosion by sulphate, due to the filling effect of the erosion products, the number of pores increased, the diameter decreased, the sphericity increased, and the stress concentration at the boundary of the pores with large sphericity rarely occurred [42]. Moreover, the shape and size of the pores were more homogeneous compared to the control, and the homogeneous pores caused an increase in the strength of the matrix. After 40 of dry–wet cycles with erosion by sulphate, due to the transitional filling of the matrix by the erosion products, matrix damage was caused, and the new pores produced at the time of damage were larger in size and number, and had poorer sphericity, which caused a high degree of stress concentration at the pore boundaries [43]. Moreover, the pores were affected by the filling of the erosion products, causing a reduction in strength.

## 4. Conclusions

This research used the uniaxial tensile test to investigate the alterations in mechanical properties of RAECC under dry–wet cyclic erosion by salt solution. Resonance frequency was employed to examine the internal integrity of the specimen, while CT scanning was used to analyze the pore structure. This study also delved into the relationship between the evolution of pores in the matrix and changes in mechanical properties during the dry–wet cyclic erosion process of salt solutions. The key findings are summarized as follows:

(1) Regarding the ultimate tensile strength of the RAECC specimens following an erosion cycle in three different solutions, the strength was generally higher than before the erosion and showed a trend of increasing first and then decreasing. The 5%Na_2_SO_4_ solution specimens, in terms of ultimate tensile strength, exhibited the most drastic changes, with a difference of 1.15 MPa between the maximum value and the minimum value. In terms of erosion severity, the order of the RAECC specimens in different solutions was as follows: 5%Na_2_SO_4_ solution > 5%Na_2_SO_4_—3%NaCl solution > aqueous solution. Moreover, the flexural strength and ultimate tensile strength of the specimens in the erosive solution changed consistently.
(2) The resonance frequency ratio showed that the internal structure of the specimen was the most complete after 15 dry–wet cycles, and the degree of completeness decreased thereafter. The X-CT test results showed that the number of cracks at the interface of the recycled fine aggregate and the substrate was high. After erosion by dry–wet sulfate cycles, the number of RAECC pores continued to increase, and the pores first decreased and then increased. Additionally, the defect volume ratio reached 9.35% after 40 dry–wet sulfate cycles, and the magnitude of the change in the pores in the two-dimensional slices after erosion was smaller than that before erosion.
(3) The process of erosion involves erosion products continuously filling and eventually destroying the pores. As erosion proceeds, the filling of pores by erosion products leads to a decrease in porosity, an increase in the number and sphericity of pores, and a tendency towards uniform pore distribution, thus increasing the strength of the matrix. With the overfilling of erosion products, the pore structure is destroyed, the porosity increases, the destruction produces a large number of pores, the sphericity is poor, and the pore destruction and the uniformity of the pore distribution decreases, which together lead to the reduction in matrix strength.

However, for the specimens tested by X-CT scanning in this paper, different specimens under the same batch were used, which may affect the pore results. Follow-up studies should further investigate multiple X-CT scans of a single specimen under different erosion cycles to eliminate the discrepancies caused by different specimens.

## Figures and Tables

**Table 1:** PE fiber performance parameters.

Density/(g/cm^3^)	Tensile Strength/MPa	Elastic Modulus/GPa	Ultimate Elongation/%	Length/mm	Diameter/μm
0.97	3000	120	5	12	24

**Table 2:** PE-ECC mix ratio. unit: kg/m^3^.

Cement	Fly Ash	Natural Sand	Aeolian Sand	RA	Water	Fiber	Superplasticizer	Thickener	Defoamer
1050	263	193	97	193	332	14.5	7.88	0.55	2.10

**Table 3:** Structural composition of X-CT pores.

Statistical Project	Unit	Results		
		C	S-20	S-40
Number of pores	Count	118,245	150,747	212,230
Material volume	mm^3^	92.47	91.045	88.184
Defect volume	mm^3^	5.91	6.231	9.093
Defect volume ratio	%	6.01	6.41	9.35
Maximum pore volume	mm^3^	3.286	5.302	6.765
Except for the defect volume of maximum pore	mm^3^	2.624	0.929	2.328
The percentage of maximum pores to total defects	%	55.60	85.09	74.40

## Data Availability

The data presented in this study are available on request from the corresponding author. The data are not publicly available due to privacy.
